# The gut-thyroid axis: physiological regulation of barrier function, microbiota, endocrine signaling and the consequences on energy metabolism

**DOI:** 10.3389/fphys.2026.1753136

**Published:** 2026-03-11

**Authors:** Lucia Acampora, Federica Restolfer, Pierluigi De Pierro, Maria Masulli, Monica Dentice, Giovanni Sarnelli, Annunziata Gaetana Cicatiello

**Affiliations:** Department of Clinical Medicine and Surgery, University of Naples Federico II, Naples, Italy

**Keywords:** epithelial permeability, gut-thyroid axis, intestinal barrier, iodine, levothyroxine absorption, microbiota, selenium, thyroid hormones

## Abstract

The gut-thyroid axis is a bidirectional physiological network in which intestinal barrier function, microbiota composition, micronutrient absorption, and Thyroid Hormone (TH) homeostasis are closely interconnected. Growing evidence indicates that alterations in intestinal integrity and microbial metabolism can significantly influence TH bioavailability and systemic endocrine regulation, while THs themselves actively shape intestinal structure and function. In this review, we summarize current knowledge on the physiological mechanisms underlying the gut–thyroid crosstalk. We first describe the organization of the intestinal barrier, focusing on epithelial transporters, tight junction dynamics, immune–epithelial interactions, and their role in controlling permeability and nutrient absorption. We then discuss how THs, via TRα1 signaling, regulate intestinal epithelial differentiation, stem-cell activity, barrier maintenance, and innate immune defenses, including the induction of intestinal alkaline phosphatase. Conversely, we examine how the intestine contributes to TH homeostasis by modulating hormone absorption, transporter-mediated uptake, deiodinase-dependent activation and inactivation, microbial deconjugation, and enterohepatic recycling. We also review the intestinal handling of iodine and selenium, emphasizing how epithelial and microbial mechanisms influence TH synthesis and peripheral metabolism. Finally, we integrate these processes into a systemic framework linking gut–thyroid interactions to energy metabolism, inflammatory status, and metabolic flexibility. Overall, this review delineates the gut–thyroid axis as a key physiological interface coordinating endocrine and gastrointestinal function and discusses emerging perspectives for therapeutic strategies targeting intestinal health to optimize TH action.

## 1 Introduction

### 1.1 The intestinal barrier: structure and mechanisms

The intestinal barrier represents a highly specialized and dynamic interface that ensures selective permeability between the external luminal environment and the internal milieu, thereby protecting the host from pathogens, toxins, and antigens while allowing efficient absorption of nutrients, water, and electrolytes (Aburto and Cryan, 2024). Structurally, the barrier is organized as a multilayered system comprising the mucus layer, the epithelial lining, and the underlying immune compartment associated with Gut-Associated Lymphoid Tissue (GALT) (Di Vincenzo et al., 2024; Paone and Cani, 2020; Vicentini et al., 2021). The transport across the enterocyte monolayer is mediated by transcellular pathway, consisting of both active and passive transport and the paracellular pathway, based on passive diffusion alongside a concentration gradient in the space between cells and it is regulated by Tight Junctions (TJs). TJs are multi-complex proteins, e.g., Claudins, Occludins, Tricellulin and Junctional Adhesion Molecules (JAMs), anchored to the cytoskeleton via zonula occludens (ZO) proteins as ZO-1 and ZO-2 and situated at the apical side of the cell (Groschw et al., 2009; Catalioto et al., 2011). Adherent junctions and desmosomes further reinforce epithelial cohesion and mechanical stability (Odenwald and Turner, 2017). Together, these structural components ensure controlled permeability and represent the primary gatekeeper of luminal–host interactions.

The gut microbiota is a fundamental regulator of intestinal barrier integrity and epithelial homeostasis. Under physiological conditions, commensal microorganisms interact with epithelial and immune cells to promote mucus production, TJ organization, and epithelial renewal (Di Vincenzo et al., 2024; Paone and Cani, 2020). A key mechanism underlying this regulation is the production of microbial metabolites, particularly short-chain fatty acids (SCFAs) such as butyrate, propionate, and acetate, which serve as energy substrates for colonocytes and act as signaling molecules that enhance TJ protein expression and mucin secretion (Vancamelbeke and Vermeire, 2017; Miner-Williams and Moughan, 2016). Conversely, alterations in microbiota composition (dysbiosis) disrupt these protective mechanisms. Loss of SCFA-producing bacteria and enrichment of pro-inflammatory taxa are associated with TJ disassembly, thinning of the mucus layer, and increased paracellular permeability (Groschw et al., 2009; Camilleri et al., 2012).

Emerging evidence demonstrates that the endocannabinoid system (ECS) is a key modulator of epithelial barrier plasticity. Activation of CB1 and CB2 receptors on intestinal epithelial cells rapidly alters TJ composition and claudin-2 localization, modifying small-intestinal permeability in response to diet or inflammation (Cuddihey et al., 2022a; Cuddihey et al., 2022b). Pharmacological modulation of these receptors induces bidirectional changes in permeability depending on baseline tone and inflammatory context, positioning the ECS as a central regulatory node linking environmental cues, barrier function, and drug absorption.

These factors converge on epithelial junctional complexes and immune–epithelial communication, establishing dysbiosis as a major determinant of intestinal permeability. Impaired intestinal barrier function has consequences that extend beyond the gastrointestinal tract.

Importantly, intestinal barrier integrity is not only shaped by microbial and environmental factors but is also regulated by systemic hormones, including Thyroid Hormones (THs), which influence epithelial renewal and TJ organization.

### 1.2 Intestinal barrier impairment and pathophysiological implications

**FIGURE 1:**
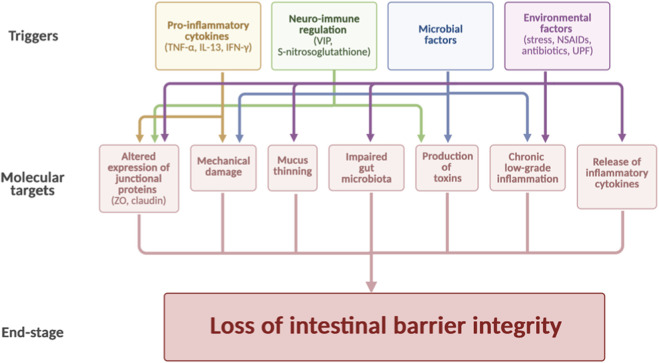
Molecular basis of the connection between various pathophysiological conditions and loss of barrier integrity. Inflammation, neuro–immune dysregulation, dietary factors, drugs, infections, and microbiota alterations converge on epithelial signaling pathways that regulate Tight Junctions (TJs) assembly and mucosal integrity. Pro-inflammatory cytokines (e.g., TNF-α, IL-6), endotoxins (LPS), and stress-related mediators activate intracellular cascades such as NF-κB and MLCK, leading to Claudin redistribution, Occludin internalization, and disruption of Zonula Occludens protein (ZO-1) anchoring to the cytoskeleton. Microbiota-derived metabolites modulate epithelial signaling through Short Chain Fatty Acids (SCFA)-dependent HDAC inhibition and GPCR activation, whereas dysbiosis promotes junctional disassembly and increased paracellular permeability. The schematic highlights the mechanistic nodes, TJ complexes, immune–epithelial crosstalk, microbial metabolites, and intracellular signaling pathways, that drive pathological barrier permeabilization.

Numerous stimuli influence intestinal barrier permeability (Figure 1). Increased permeability allows luminal antigens and microbial-derived products, including lipopolysaccharide (LPS), to translocate into the lamina propria and systemic circulation, triggering chronic low-grade inflammation (Di Vincenzo et al., 2024; Camilleri et al., 2012). This phenomenon has been implicated in the pathogenesis of inflammatory, autoimmune, functional, and endocrine–metabolic disorders (Dunleavy et al., 2023; Tristan Asensi et al., 2023). Clinical conditions such as Crohn’s disease (CD), ulcerative colitis (UC), celiac disease (CeD), and irritable bowel syndrome (IBS) are characterized by altered TJ expression and epithelial damage, which correlate with disease severity and systemic manifestations (Camilleri et al., 2012; Dunleavy et al., 2023; Tristan Asensi et al., 2023). Barrier impairment contributes to metabolic dysregulation by promoting inflammatory signaling that interferes with insulin sensitivity, mitochondrial function, and energy expenditure (Vancamelbeke and Vermeire, 2017; Miner-Williams and Moughan, 2016).

Cytokines, such as Tumor Necrosis Factor alpha (TNF-α), are responsible for the loss of function of TJ-associated proteins, allowing detrimental substances to pass through the epithelium, whereas Interleukin 13 (IL-13) may promote claudin-2 upregulation, a condition associated with increased intestinal permeability (Camilleri et al., 2012). A diverse array of inflammatory mediators, including interleukins IL-4, IL-6, IL-22, and Interferon gamma (IFN-γ), as well as microbial-derived signals, contribute to TJs modulation (Camilleri et al., 2012).

Chronic stress, genetics, infections, nonsteroidal anti-inflammatory drugs (NSAIDs), antibiotics, and diets rich in ultra-processed foods (UPFs) have a negative impact on barrier function (Macura et al., 2024). CeD is driven by gluten ingestion in patients with a genetic predisposition. Gliadin, by binding to CXCR3 on the apical surface of intestinal epithelial cells, may promote the release of zonulin, a protein that regulates the paracellular transport pathway and is highly expressed in CeD. Immune activation and CD8-mediated killing result in villous atrophy and crypt hyperplasia, which impair both barrier structural integrity and its absorptive function (Miner-Williams and Moughan, 2016).

Table 1 describes different pathophysiological alterations and mechanisms underlying intestinal permeability and affecting systemic metabolism (Lee et al., 2020; Dutt et al., 2025). As also described below, barrier impairment not only contributes to gastrointestinal diseases, but may also have systemic effects, including the absorption of THs, iodine and thyroid-related substances.

**TABLE 1:** Pathophysiological alterations and mechanisms associated with increased intestinal permeability and their impact on systemic metabolism.

Pathogenetic mechanism	Disease	Effect on systemic metabolism
Junctional proteins dysfunction and epithelial damage (Cuddihey et al., 2022a; Jin et al., 2022)	IBS, UC, CD, CeD	Low-grade inflammation, impaired insulin sensitivity
Microbial translocation (Arpaia et al., 2013)	UC, CD, CeD	Systemic inflammation, increased oxidative stress
Aberrant immune response to dietary and microbial antigens (Cuddihey et al., 2022a)	UC, CD, CeD	Altered energy expenditure and substrate utilization
Loss of absorptive surface (e.g., villous atrophy, ulcerations) (Dutt et al., 2025)	UC, CD, CeD	Macro/micronutrient malabsorption, weight loss
Gut microbiota dysbiosis and impaired microbial-epithelial interactions (Cuddihey et al., 2022b)	IBS, UC, CD, CeD	SCFAs and bile-acid imbalance, increased obesity and insulin-resistance risk
Altered water and electrolyte transport (Aburto and Cryan, 2024; Odenwald and Turner, 2017)	IBS, UC, CD, CeD	Fluid-electrolyte imbalance, impaired nutrient uptake; fatigue
Chronic low-grade mucosal inflammation (Tristan Asensi et al., 2023; Macura et al., 2024)	IBS, UC, CD	Systemic low-grade inflammation, insulin resistance
Visceral hypersensitivity and brain–gut axis impairment (Miner-Williams and Moughan, 2016)	IBS	Stresss-axis activation, food intake dysregulation, altered energy balance
Genetic susceptibility (e.g., HLA-DQ2/DQ8, NOD2, IL23R) (Camilleri et al., 2012; Lee et al., 2020; Dutt et al., 2025; Ebert, 2010)	UC, CD, CeD	Higher lifetime risk of malabsorption, micronutrient deficits, and metabolic diseases

CD, Chron’s disease; UC, ulcerative colitis; CeD, celiac disease; IBS, irritable bowel syndrome.

### 1.3 Biosynthesis, secretion, and endocrine regulation of thyroid hormones

The thyroid synthesizes, stores, and secretes the iodothyronines T4 and T3, which are released to the circulation after pituitary Thyroid Stimulating Hormone (TSH) stimulated endocytosis and lysosomal proteolysis of Tg (Jing and Zhang, 2022). THs play multiple roles, supporting processes such as proliferation, cell differentiation, thermoregulation, metabolism, and energy production, thus contributing to growth and development (Cicatiello et al., 2022; Luongo et al., 2019).

Systemic control is provided by the hypothalamic-pituitary-thyroid (HPT) axis. Hypothalamic Thyrotropin Releasing Hormone (TRH) stimulates TSH secretion, which in turn drives TH production and release (predominantly T4, with a smaller fraction of T3); circulating THs exert negative feedback on both hypothalamus and pituitary to maintain homeostasis (Feldt-Rasmussen et al., 2021). T3 is the biologically more potent hormone with a short plasma half-life (∼1 day), whereas T4 has a longer half-life (∼7 days) and serves largely as a prohormone for peripheral T3 generation (Nicoloff et al., 1972).

**FIGURE 2:**
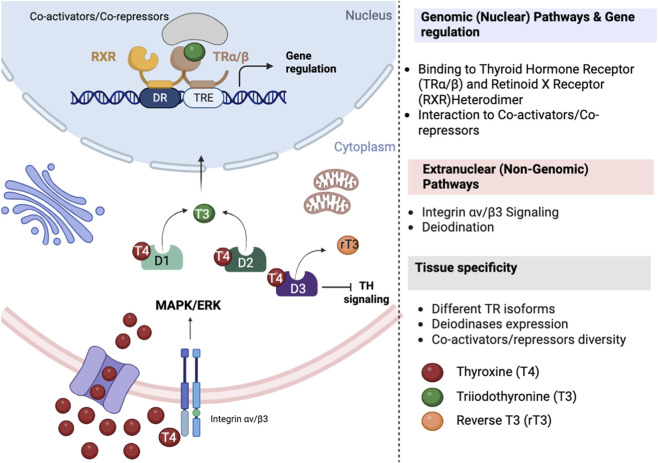
Extranuclear and nuclear Thyroid Hormone pathways. Thyroid hormone (TH) signaling is regulated at multiple intracellular levels. In the genomic pathway, T3 enters the nucleus via specific transporters (MCT8, OATPs, LATs) and binds Thyroid Hormone Receptor (TRα or TRβ), which heterodimerize with RXR and interact with TH Response Elements (TREs), recruiting co-activators or co-repressors to regulate transcription of metabolic genes. In parallel, non-genomic signaling is initiated at the plasma membrane, notably via integrin αvβ3, where T4 binding activates MAPK/ERK and PI3K pathways, modulating proliferation, mitochondrial activity, and cellular metabolism independently of direct transcriptional regulation. Intracellular TH availability is further controlled by deiodinases (D1, D2, D3), which locally activate or inactivate T4 and T3, creating tissue-specific gradients of TH signaling. Differential expression of transporters, receptors, deiodinases, and coregulators determines cell-type–specific TH responsiveness.

Cellular entry of THs requires specific transporters whose tissue distribution shapes local hormone bioavailability (Figure 2). Monocarboxylate Transporter 8 (MCT8) transports both T3 and T4 with high efficiency and is critical at the blood-brain barrier; Organic Anion-Transporting Polypeptide 1C1 (OATP1C1) preferentially transports T4 and 3,3′,5′-triiodothyronine, commonly referred to as reverse triiodothyronine (rT3) into the brain; Monocarboxylate Transporter 10 (MCT10) also facilitates T3 transport in several tissues. Pathogenic variants of MCT8 and alterations in OATP1C1 highlight the physiological importance of transporter-mediated TH flux (Bianco et al., 2019).

Peripheral metabolism is governed by the selenoprotein deiodinases. Deiodinase type 1 (D1) and Deiodinase type 2 (D2) catalyze outer-ring deiodination of T4 to produce active T3, thereby sustaining systemic and tissue-specific T3 availability (Miro et al., 2025) (Figure 2). Deiodinase type 3 (D3) inactivates THs by inner-ring deiodination (T4 into rT3; T3 into T2), providing a brake on signaling during development, illness, and in selected tissues (e.g., placenta, brain, skin). The coordinated expression of transporters and deiodinases creates microenvironments in which local genomic TH signaling may diverge from serum concentrations (Sabatino et al., 2021; Ambrosio et al., 2013; Miro et al., 2023). Accordingly, deiodinases altered function leads to tissue-specific THs homeostasis with consequences on whole body energy metabolism (Miro et al., 2025; Cicatiello et al., 2018), as well as to dysregulated cell proliferation and differentiation, augmenting pathogenic consequences as inflammation and tumorigenic risks (Sagliocchi et al., 2023a; Nappi et al., 2025a; Sagliocchi et al., 2023b).

THs regulate gene expression via nuclear thyroid hormone receptors (TRα encoded by THRA gene and TRβ encoded by THRB gene), which bind DNA, typically as RXR heterodimers, on thyroid hormone response elements (TRE) to recruit corepressors or coactivators in a ligand-dependent manner; this “coregulator switch” underlies gene and context-specific activation or repression (Sinha and Yen, 2024; Nappi et al., 2022). In parallel, non-genomic actions initiated at the plasma membrane, most notably via integrin αvβ3, rapidly activate signaling cascades (e.g., ERK1/2, PI3K/MAPK, STAT), illustrating mechanistic crosstalk between extranuclear and nuclear pathways and providing a rationale for tissue-specific responses to THs (Davis et al., 2008; Nappi et al., 2021).

Beyond T3 and T4, rT3 is a naturally occurring TH metabolites generated by inner ring deiodination of T4 by D3 that possess biological activities and mediate metabolic effects. Although rT3 lacks significant affinity for nuclear TH receptors and does not activate canonical TH-dependent gene transcription, it exerts important metabolic effects by limiting intracellular T3 availability.

### 1.4 The gut-thyroid axis: functional interdependence and systemic crosstalk

Systemically, the gut is a critical hub for peripheral TH metabolism. Although different mechanisms of systemic and peripheral regulation of THs have been described, additional metabolic processes, including conjugation, also contribute to TH homeostasis. Hepatic conjugation (glucuronidation, sulfation) generates T3G and T4G from T3 and T4; in the intestinal lumen, microbial β-glucuronidases and sulfatases can deconjugate these metabolites, regenerating free iodothyronines and allowing enterohepatic recycling (Hazenberg et al., 1988). Consequently, the composition of the gut microbiota and the state of the barrier can modulate the availability of circulating and tissue TH levels by altering the efficiency of deconjugation. An imbalance in gut microbial composition has been observed not only in Auto-Immune Thyroid Diseases (AITD) but also in thyroid carcinoma with a higher number of pro-inflammatory bacterial metabolites. On the other hand, the microbiota also affects the availability of essential micronutrients necessary for the functionality of the thyroid gland (Knezevic et al., 2020). In addition, as described in the next paragraph, the intestine regulates TH homeostasis by modulating T3 and T4 absorption, with a critical impact on TH-dependent efficiency in peripheral tissues (Virili et al., 2019).

Viceversa, THs are active regulators of intestinal development and epithelial maintenance. In the intestine, the predominant isoform of the nuclear receptor is TRα1, which orchestrates proliferation-differentiation programs in crypt-villous units by interfacing with the Wnt/β-catenin pathway; genetic and mechanistic studies demonstrate that T3-TRα1 directly controls β-catenin and cell cycle genes (e.g., D-type cyclins and MYC), thus supporting epithelial renewal (Plateroti et al., 2006). This TRα1 control is conserved across species and remains operative in the adult intestine, confirming the intestine as a true target organ for TH (Plateroti et al., 2006). In addition to morphogenesis, THs contribute to barrier integrity and mucosal differentiation through the transcriptional regulation of epithelial effectors. A typical example is IALP, a differentiation-dependent brush border enzyme whose expression increases during enterocyte maturation (Hodin et al., 1994), which is a T3-responsive gene (Malo et al., 2004).

Importantly, THs also modulate gastrointestinal physiology by regulating intestinal motility, gastric secretion, and absorptive function (Ebert, 2010). TH excess is associated with accelerated gastric emptying and increased intestinal transit, whereas hypothyroidism commonly leads to delayed gastric emptying, reduced peristalsis, and constipation (Ishikawa and Raskin, 1995). These effects are mediated through both genomic and non-genomic mechanisms, including modulation of smooth muscle contractility, enteric nervous system activity, and ion channel expression in intestinal cells (Ishikawa and Raskin, 1995). Moreover, THs influence gastric acid secretion and digestive enzyme activity, thereby indirectly affecting nutrient bioavailability and drug absorption. Collectively, these actions position THs as central regulators of gastrointestinal function, linking endocrine status to intestinal motility, secretory dynamics, and absorptive capacity.

As a result of the tight Gut-Thyroid interconnection, multiple intestinal alteration can profoundly affect the TH homeostasis and lead to TH dysfunctions. For instance, specific SCFAs exert distinct roles in thyroid function and diseases. Butyric acid produced by *F. prausnitzii* regulates NIS expression in thyroid cells through histone deacetylase (HDAC) -dependent epigenetic mechanism. It can inhibit HDAC1 and activate NIS re-expression in thyroid cancer cells, thereby inducing re-differentiation and iodine uptake (Zhou et al., 2018) (Rathod et al., 2020). In addition to butyrate, propionate but not acetate facilitates extra thymic *de novo* Treg-cell generation (Arpaia et al., 2013). These SCFAs mediate a crosstalk between the commensal microbiota and the immune system, influencing the balance between pro- and anti-inflammatory mechanisms.

Moreover, distinct microbial signatures have been identified across different thyroid pathologies. In Hashimoto’s thyroiditis, the gut microbiome is characterized by a reduction in *Bifidobacterium* (Gram-positive, LPS-negative) and a concomitant increase in *Bacteroides* (Gram-negative bacteria, LPS positive) (Fang and Ning, 2024; Pagliuca et al., 2016). Thus, the microbial shift involves both loss of beneficial LPS-negative taxa and enrichment of LPS-containing organisms. In Graves’ disease, a decrease in beneficial *Firmicutes* such as *Faecalibacterium* and is observed alongside an increased abundance of Gram-negative taxa, including *Bacteroides*, *Enterobacter*, and *Chryseobacterium*, all of which contain LPS in their outer membrane. Notably, some studies also report enrichment of *Prevotella*, further supporting a shift toward endotoxin-containing microbial communities (Jiang et al., 2021; Zheng et al., 2023). Non-autoimmune thyroid diseases also exhibit distinct patterns. Thyroid nodules are associated primarily with a reduction in butyrate-producing genera such as *Butyrivibrio* and *Coprococcus*, suggesting loss of anti-inflammatory capacity rather than exclusive enrichment of LPS producers. In thyroid cancer, depletion of *Christensenellaceae* and *Eubacterium* is accompanied by a relative increase in Gram-negative bacteria, including *Bacteroides*, potentially contributing to a pro-inflammatory microenvironment.

Collectively, these findings indicate that thyroid-associated dysbiosis reflects a combined pattern of enrichment of Gram-negative, LPS-containing bacteria and depletion of beneficial Gram-positive, SCFAs–producing taxa.

## 2 Intestinal barrier and T4 bioavailability

**FIGURE 3:**
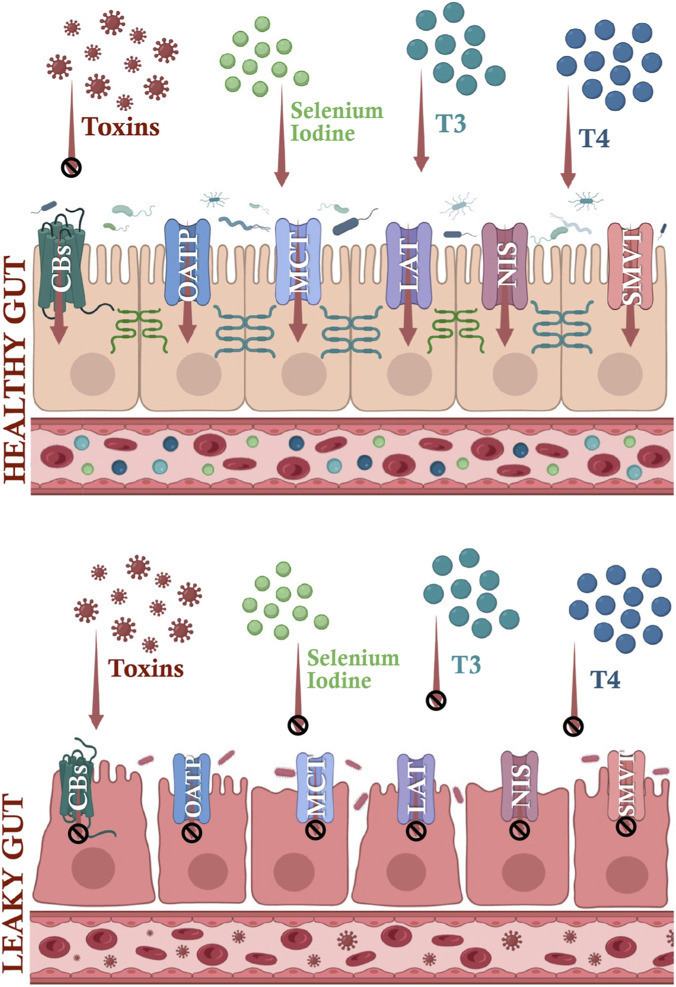
Intestinal barrier integrity shapes the uptake of Thyroid Hormones and related micronutrients. Upper panel: physiological barrier state. Intact Tight Junctions (TJs) maintain controlled paracellular permeability, while transcellular uptake of T3, T4, iodide, and selenium occurs through specific apical transporters including OATPs, MCT8/MCT10, LAT1/2, NIS, and SMVT. Coordinated epithelial transport ensures systemic delivery of thyroid hormones and essential cofactors required for thyroid hormone synthesis and deiodinase activity. Lower panel: disrupted barrier state (“leaky gut”). TJ disassembly and epithelial stress increase paracellular permeability to luminal toxins (e.g., LPS) while impairing regulated transcellular transport of T3, T4, iodide, and selenium. Barrier dysfunction is associated with inflammatory signaling, reduced transporter efficiency, and altered micronutrient handling, collectively diminishing thyroid hormone bioavailability. Arrows indicate effective transport; Ø indicates reduced transport capacity and functional impairment.

Levothyroxine (L-T4) is absorbed primarily in the small intestine, particularly in the duodenum and ileum (Virili et al., 2020). Under fasting conditions, approximately 60%–80% of an administered dose is absorbed, depending on formulation (Ghosh et al., 2020). Absorption efficiency is influenced by gastric pH, intestinal motility, mucosal surface area, and luminal composition (Wiesner et al., 2021; Liu et al., 2023). Conditions associated with mucosal injury or malabsorption can significantly reduce T4 uptake and bioavailability, thereby increasing L-T4 dose requirements (De Carvalho et al., 2018) (Figure 3).

Although early pharmacokinetic models proposed passive diffusion, molecular studies demonstrate that T4 uptake across enterocytes is largely transporter-mediated. Identified carriers include Organic Anion Transporting Polypeptides (OATP1A2, OATP1A5), Monocarboxylate Transporters (MCT8, MCT10), L-type Amino Acid Transporters (LAT1/2), and additional members of the solute carrier (SLC) family (Teumer et al., 2018; Meyer Zu Schwabedissen et al., 2018; Friesema et al., 2008). These transporters facilitate transcellular T4 movement from the intestinal lumen into enterocytes in a partially redundant but regulated manner. Importantly, their expression and activity may be altered under inflammatory or dysbiotic conditions, thereby modulating systemic hormone exposure independently of epithelial barrier integrity.

In addition, disruption of TJ architecture—as observed in IBD, CeD, and other enteropathies—may impair T4 bioavailability by destabilizing the absorptive epithelium and altering mucosal permeability (De Carvalho et al., 2018). Biomarkers of barrier dysfunction, such as ZO-1 and LPS-binding protein, have been associated with autoimmune thyroid disease severity (Benvenga et al., 2018), supporting the concept that epithelial barrier alterations may contribute to impaired hormone handling and dose instability.

Experimental evidence supports a causal role of microbial composition in systemic TH regulation. Fecal microbiota transplantation from individuals with primary hypothyroidism into mice resulted in altered circulating total T4 levels compared with mice receiving microbiota from healthy donors (Fang and Ning, 2024), suggesting that dysbiotic communities can influence hormone homeostasis. Patients with IBD, IBS, liver cirrhosis, or systemic sclerosis frequently require higher L-T4 doses, likely reflecting impaired absorption and altered pharmacokinetics (Virili et al., 2019; Benvenga et al., 2018; Henderson et al., 2024; Jin et al., 2022; Peng et al., 2025; Lobasso et al., 2017). Case reports describe severe hypothyroidism due to ineffective L-T4 therapy in the setting of gastrointestinal dysfunction (Lobasso et al., 2017). Although direct causality remains incompletely established, cumulative mechanistic and clinical evidence supports a clinically meaningful link between intestinal barrier integrity, transporter biology, microbiota composition, and L-T4 dose stability.

## 3 Micronutrients absorption and thyroid hormones metabolism: iodine and selenium

Micronutrient absorption within the gastrointestinal tract represents a critical determinant of TH homeostasis. Among these micronutrients, iodine and selenium occupy central roles, influencing both the synthesis and metabolism of THs as well as the integrity of the intestinal barrier itself (Knezevic et al., 2020; Clemente-Suarez et al., 2024). Iodine is an essential micronutrient required for the biosynthesis of both T4 and T3. Adequate iodine availability ensures sufficient production of these hormones (Rokita et al., 2010), whereas both deficiency and excess can perturb thyroid function. Iodine absorption across the intestinal epithelium is mediated primarily by the NIS, expressed on the apical membrane of enterocytes (Nicola et al., 2009a; Nicola et al., 2009b). Expression and activity of intestinal NIS respond dynamically to dietary iodine intake, thus maintaining systemic equilibrium and ensuring TH biosynthetic capacity (Nicola et al., 2015). In addition to NIS, the sodium-dependent multivitamin transporter (SMVT), which is classically associated with biotin and pantothenate uptake, is expressed in the intestine and multiple tissues (Quick and Shi, 2015). *In vitro* studies reveal that SMVT can mediate sodium-coupled iodide transport, suggesting a complementary mechanism for intestinal iodine uptake. Parallel evidence implicates the gut microbiota as an additional determinant of iodine bioavailability. AITD, including Hashimoto’s thyroiditis, is frequently associated with altered microbiota composition and compromised epithelial integrity (Sawicka-Gutaj et al., 2022). Both human and animal model studies demonstrate that excessive iodine intake disrupts microbial community structure, particularly by depleting butyrate-producing bacteria and SCFAs. These alterations impair T-regulatory and T-helper-17 (Treg/Th17) balance, heighten mucosal inflammation, and exacerbate autoimmune thyroid pathology (Gong et al., 2024). Conversely, antibiotic-induced dysbiosis in rats reduces iodine absorption, highlighting the microbiota’s direct contribution to micronutrient bioavailability (Vought et al., 1972).

Selenium is another essential trace element integral to TH physiology. Its biological importance arises from its incorporation into selenoproteins, including the deiodinase enzymes D1, D2, D3 (Kobayashi et al., 2021; Ventura et al., 2017). Sufficient selenium levels are therefore required to sustain optimal deiodinase activity and maintain both systemic and peripheral hormone balance (Bates et al., 2000). In selenium-deficient states, both experimental and clinical data demonstrate impaired TH regulation (Nappi et al., 2025b). Selenium-deficient mice exhibit early-onset gut barrier dysfunction and inflammation, followed by disrupted TH synthesis and aggravated autoimmunity, reinforcing the interdependence between micronutrient status, intestinal homeostasis, and endocrine health (Yan et al., 2024). Selenium’s antioxidant functions mitigate oxidative stress and apoptotic damage within the thyroid, effects particularly relevant in autoimmune thyroiditis (Huwiler et al., 2024) (Gartner et al., 2002). Epidemiological and interventional studies link low selenium status to increased risk of autoimmune thyroid diseases, including Hashimoto’s and Graves’ disease (Winther et al., 2020). Cross-sectional analyses also report lower selenium concentrations in thyroid carcinoma patients, suggesting a potential role in thyroid oncogenesis (Bates et al., 2000). Clinical trials indicate that selenium supplementation can decrease thyroid autoantibody titers and modulate immune activity, supporting its therapeutic potential in autoimmune thyroid disorders (Nettore et al., 2017). Maintaining adequate selenium intake is therefore essential for both antioxidant defense and TH metabolism.

Together, iodine and selenium exemplify how micronutrient absorption intersects with epithelial integrity, immune regulation, and microbial metabolism. Looking forward, comprehensive analyses employing single-cell transcriptomics and metagenomic profiling will be essential to define the bidirectional fluxes of micronutrients and hormones across the intestinal barrier. From a translational standpoint, targeted modulation of the microbiome, reinforcement of barrier integrity, and precision micronutrient supplementation represent promising strategies for personalized management of thyroid disorders.

Modulation of the gut microbiome can be achieved through targeted strategies including probiotic administration, prebiotic supplementation, and FMT. Probiotic supplementation has been shown to alleviate oral–gut microbiota dysbiosis and mitigate TH withdrawal–related complications in thyroid cancer patients undergoing radioiodine therapy after thyroidectomy (Lin et al., 2022). Prebiotic fibers such as inulin enhance SCFAs production, thereby supporting epithelial TJ integrity and mucosal barrier function (Sheng et al., 2023). Furthermore, FMT from individuals with primary hypothyroidism in mice resulted in altered circulating TH levels compared to those receiving microbiota from healthy donors, supporting a causal role of gut dysbiosis in TH regulation (Su et al., 2020). Collectively, these findings provide mechanistically grounded approaches for the personalized management of thyroid disorders through microbiome- and barrier-targeted strategies.

## 4 Systemic metabolic effects of gut-thyroid crosstalk

**FIGURE 4:**
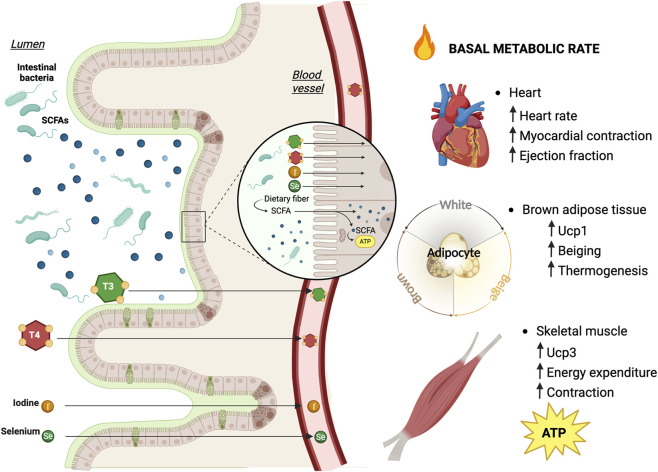
Intestinal barrier integrity as a central determinant of Thyroid Hormone bioavailability and peripheral metabolic function. An intact intestinal barrier is essential for efficient absorption of thyroid hormones (T3 and T4) and of key micronutrients, including iodine and selenium, which are required for thyroid hormone synthesis and deiodinase activity. Proper epithelial transport, Tight Junction (TJ) organization, and luminal homeostasis collectively ensure optimal thyroid hormone bioavailability. Adequate systemic absorption of T3 and T4 guarantees their appropriate delivery to peripheral target organs such as the heart, Brown Adipose Tissue (BAT), and skeletal muscle, where thyroid hormone signaling regulates mitochondrial oxidative phosphorylation, thermogenesis, and overall energy metabolism. Disruption of barrier integrity may therefore impair hormone absorption and micronutrient availability, ultimately compromising peripheral thyroid hormone action and metabolic homeostasis.

The gut-thyroid axis plays a central role in integrating endocrine signals with whole-body metabolic regulation. Under physiological conditions, THs act as key determinants of basal metabolic rate, mitochondrial respiration, lipid turnover, and glucose utilization, while the intestinal barrier and the gut microbiota influence TH bioactivation, transport, and tissue availability (Miro et al., 2025). This interdependence establishes a homeostatic metabolic loop in which endocrine and gastrointestinal systems continually adjust their outputs to maintain energetic balance (Figure 4) (Virili et al., 2019; De Vadder et al., 2014).

A central convergence point of this axis is the regulation of local T3 production through deiodinases. Microbiota-derived metabolites, particularly SCFAs such as butyrate and propionate, enhance mitochondrial oxidative capacity and insulin sensitivity while simultaneously modulating the expression and activity of deiodinases D1 and D2. By promoting peripheral conversion of T4 to bioactive T3, SCFAs directly amplify TH signaling in metabolically active tissues.

Bile acids represent an additional mechanistic bridge between the gut and systemic TH action. Primary bile acids synthesized in the liver are converted by intestinal microbiota into secondary bile acids, including deoxycholic acid (DCA) and lithocholic acid (LCA) (Thomas et al., 2008). These microbiota-derived species are potent agonists of the G protein–coupled bile acid receptor TGR5 (Pathak et al., 2018). Upon activation, TGR5 stimulates Gαs-dependent adenylate cyclase activity, increasing intracellular cAMP, activating PKA, and promoting CREB phosphorylation. Phosphorylated CREB enhances transcription of D2, thereby increasing intracellular T3 production (Watanabe et al., 2006). In turn, increased D2 expression augments local T3 availability in skeletal muscle and brown adipose tissue, promoting mitochondrial oxidative phosphorylation, thermogenic uncoupling, and energy expenditure (Watanabe et al., 2006).

Barrier integrity further modulates this axis. Increased intestinal permeability facilitates translocation of endotoxins such as LPS, promoting chronic low-grade inflammation (Cani et al., 2007). Inflammatory signaling interferes with TH receptor function and mitochondrial responsiveness to T3, thereby attenuating hormone-driven metabolic processes. In parallel, impaired iodine and selenium absorption compromises TH synthesis and deiodinase activity, reducing systemic and tissue-specific TH availability.

Thus, gut-derived metabolites, bile acid signaling, barrier integrity, and micronutrient absorption converge on a shared regulatory node: intracellular T3 generation and thyroid receptor activation. Disruption of this integrated axis—whether through dysbiosis, inflammation, or thyroid dysfunction—produces coordinated alterations in mitochondrial efficiency, substrate utilization, and thermogenesis, contributing to obesity, metabolic syndrome, and systemic energy imbalance.

Collectively, these findings support a unified gut–microbiota–thyroid–tissue axis in which intestinal physiology governs peripheral TH activation, and TH signaling, in turn, shapes systemic metabolic phenotype.

Together, these findings position the gut-thyroid-microbiota triad as a crucial physiological axis governing metabolic equilibrium. Understanding the metabolic implications of this inter-organ communication may open new therapeutic perspectives, including microbiota-modulating strategies, barrier-restoring interventions, and tailored micronutrient supplementation, with the potential to improve metabolic and endocrine outcomes simultaneously.

## 5 Conclusion

The gut-thyroid axis represents a crucial physiological interface integrating epithelial, microbial, and endocrine signaling. Its disruption contributes to thyroid dysfunction, altered drug absorption, and systemic inflammation. By integrating evidence on epithelial barrier integrity, transporter expression, microbiota-derived metabolites, and micronutrient handling with studies of TH action on both intestinal structure and function, it emerges that gut-thyroid crosstalk shapes systemic energy expenditure, thermogenesis and glucose-lipid homeostasis. Recognizing the TH-gut axis as a metabolic hub opens new avenues for interventions that simultaneously target thyroid function, barrier health, microbiota composition, and micronutrient status to optimize whole-body metabolic homeostasis.
